# Short-term balance training and acute effects on postural sway in balance-deficient older adults: a randomized controlled trial

**DOI:** 10.1186/s13102-021-00251-x

**Published:** 2021-03-09

**Authors:** Niklas Sörlén, Andreas Hult, Peter Nordström, Anna Nordström, Jonas Johansson

**Affiliations:** 1 Department of Clinical Science, Neurosciences, Umeå University, Umeå, Sweden; 2 Department of Public Health and Clinical Medicine, Section for Sustainable Health, Umeå University, Umeå, Sweden; 3 Department of Community Medicine and Rehabilitation, Section for Sports Medicine, Umeå University, Umeå, Sweden; 4 Umeå School of Sport Sciences, Umeå University, Umeå, Sweden; 5 Department of Community Medicine and Rehabilitation, Geriatric Medicine, Umeå University, Umeå, Sweden; 6 School of Sport Sciences, UiT The Arctic University of Norway, Tromsø, Norway; 7 Department of Community Medicine, UiT The Arctic University of Norway, Tromsø, Norway

**Keywords:** Postural control, Older adults, RCT, Balance exercise

## Abstract

**Background:**

We aimed to determine the effectiveness of 4 weeks of balance exercise compared with no intervention on objectively measured postural sway.

**Methods:**

This was a single-center parallel randomized controlled, open label, trial. A six-sided dice was used for allocation at a 1:1-ratio between exercise and control. The trial was performed at a university hospital clinic in Sweden and recruited community-dwelling older adults with documented postural instability. The intervention consisted of progressively challenging balance exercise three times per week, during 4 weeks, with follow-up at week five. Main outcome measures were objective postural sway length during eyes open and eyes closed conditions.

**Results:**

Sixty-five participants aged 70 years (balance exercise *n* = 32; no intervention *n* = 33) were randomized. 14 participants were excluded from analysis because of early dropout before follow-up at week five, leaving 51 (*n* = 22; *n* = 29) participants for analysis. No significant differences were detected between the groups in any of the postural sway outcomes. Within-group analyses showed significant improvements in hand grip strength for the intervention group, while Timed Up & Go improvements were comparable between groups but only statistically significant in the control group.

**Conclusions:**

Performing balance exercise over a four-week intervention period did not acutely improve postural sway in balance-deficient older adults. The lower limit in duration and frequency to achieve positive effects remains unclear.

**Trial registration:**

Clinical trials NCT03227666, July 24, 2017, retrospectively registered.

**Supplementary Information:**

The online version contains supplementary material available at 10.1186/s13102-021-00251-x.

## Introduction

Today, fracture prevention is primarily focused on osteoporosis and bone-strengthening measures [1]. However, research over the past decades have shown that only 20% of fracture patients have osteoporosis while 98% of low-energy fractures are caused by a fall [2, 3]. The knowledge is currently evolving regarding risk factors for fractures and, more importantly, falling. Impairments in balance, functional mobility, gait, and muscle strength have shown to be important physical risk factors for falls among older adults [4–9]. Furthermore, psychological aspects such as fear of falling and poor self-efficacy are also known to predict falls [10–12]. It is estimated that two thirds of deaths due to fall accidents could potentially be prevented, either through balance and strength exercise, or through an attenuation of extrinsic or intrinsic risk factors [8].

Another risk factor that has emerged as a potential modifiable factor is reduced postural sway [9, 13–16]. Postural sway is the result of the human body’s continuous adjustments to uphold postural stability during an upright stance, and can be assessed by measuring the deviations in the center of pressure location on the supporting surface by means of a force platform. In a recent population-based study with 1877 older adults, it was shown that persons in the highest quintile of postural sway had a 75–90% increased risk of incident falls, signifying that these objective measures independently predict falls in elderly [9]. It is acknowledged that postural sway may be modifiable with the use of specific training, such as balance exercise [16–19], and while as little as 3–6 weeks of training can produce significant results, 11–12 weeks seems to be the most effective duration [20, 21]. However, it may be of interest, from a cost-effective standpoint, to explore whether an exercise program with a shorter time window could induce relevant effects, especially in older individuals with documented poor balance. To support this, a recent study showed that 4 weeks of resistance exercise was sufficient to increase muscle mass and physical function in untrained individuals, a group that can experience relatively quick improvements according to general exercise principles [22, 23].

Thus, the purpose of this study was to investigate whether 4 weeks of progressively challenging, low-threshold balance training was sufficient as a single intervention to improve postural sway and other risk factors for falling in individuals with detected postural instability. The primary outcome was change in postural sway length. Secondary outcomes were Timed Up & Go (TUG) time, handgrip strength and score on the Falls Efficacy Scale (FES) and Falls Efficacy Scale-International (FES-I) questionnaires.

## Methods

The present study was a two-armed, parallel randomized controlled trial, conducted at a university hospital clinic in northern Sweden. The trial was registered at clinicaltrials.gov (NCT03227666), approved by the Umeå University research ethics committee, and conducted in accordance with the Declaration of Helsinki. All participants provided informed written consent. Initial power calculations were based on earlier bipedal postural sway measurements performed by individuals within the inclusion criteria (eyes open trial, mean 528 mm ± 179 mm). Assuming a power of 80% with α < 0.05, a sample size of 58 participants was deemed necessary to detect ~ 130 mm difference in postural sway between the groups, a feasible change after 6 weeks as documented by a previous balance exercise intervention [24].

### Participants

The present trial recruited participants from an ongoing population-based primary prevention study investigating risk factors for non-communicable diseases. For inclusion in the original primary prevention study, participants had to be living in the Umeå municipality and be 70 years of age at the time of examination. Details of the study procedure have been described previously [9].

Eligibility criteria for the present trial were postural sway length with eyes open (PS_EO_) ≥ 400 mm or postural sway length with eyes closed (PS_EC_) ≥ 920 mm, and being able to walk without a walking aid. The postural sway length criteria were based on previous fall-risk analyses of the same cohort as the present sample was drawn from, representing the highest quintiles of PS_EO_ or PS_EC_ [9].

Upon enrollment in the present trial (March 2017–July 2017), participants arrived at a hospital clinic in Umeå, Sweden and answered a questionnaire on their medical and accident history, and their current medications. The baseline assessments, except anthropometrics, FES and FES-I, were drawn from the previous cohort study examination and were performed within 3 months prior to each participant’s eligibility assessment and within 4 months to actual trial commencement. Follow-up assessments were performed at week five at the hospital clinic after the intervention period. The time-of-day of baseline and follow-up assessments were not standardized.

### Intervention

The intervention comprised a four-week, weekly-progressing exercise program targeting neuromuscular function and lower-extremity strength with the purpose of increasing postural control by providing gradually increased challenge to the participants’ balance ability and strengthen the involved musculature. The balance exercise program was designed for individuals with documented poor postural control and consisted of both steady-state balance exercises and dynamic strength exercises (for more information, see Additional File 1). Progression of the steady-state exercises was based on a previous study evaluating difficulties of various postural stances in older adults [25]. We incorporated dynamic exercises targeting muscles involved in the anterior-posterior and medio-lateral directions of postural control, with the purpose of strengthening the efferent response during static stance. In designing the exercise program, inspiration was drawn from previous studies and systematic reviews on balance exercise [26–30]. In short, participants progressively performed semi-tandem, tandem and one-leg stances during eyes open and eyes closed conditions throughout the four-week program. They were instructed to maintain these stances for between 10 to 30 s and to progress to a more difficult stance if they exceeded this time. In addition, they performed chair stands, lateral leg-raises and calf-raises for at least 8–12 repetitions and 3 sets. The total exercise dose was roughly 180 min, performed for approximately 15 min per session, three times weekly, during 4 weeks. The training was performed in groups of 6–8 people under supervision by an Exercise Physiologist.

Participants were instructed not to perform the exercises longer than prescribed, and rest was administered ad libitum, but not surpassing 1 min between sets. Chairs were used as support by the participants during standing exercises when or if needed. However, they were encouraged to challenge themselves by abstaining the use of support as often as possible. Individual attendance was recorded for all 12 sessions, and the data were used to calculate a mean compliance rate.

The control subjects were given no intervention or care in this trial. After the trial ended, the training program was handed out to the control group participants. It should be noted that both intervention group participants and control group participants were drawn from a cohort study, where they had received simple lifestyle advice based on their results.

### Outcomes

Follow-up assessments were made at week five. The primary outcome measure was group-difference in mean change in postural sway, recorded during bipedal static stance during 2 one-minute trials with eyes open and eyes closed, respectively. A Nintendo Wii Balance Board device labeled “Nintendo RVL-WBC-01” was used to record the center of pressure position at a sampling frequency of 100 Hz, yielding a measure of total postural sway length in millimeters. The raw signal was filtered using a 3rd degree Butterworth filter at 10 Hz. Validation and test-retest reliability has been performed on the equipment, exhibiting moderate (Intraclass-coefficient [ICC] 0.60 for PS_EO_) and excellent (ICC 0.94 for PS_EC_) reliability [9]. Postural sway measurements were performed without shoes and with a foot width of 20 cm. The participants were instructed to keep their eyes focused straight forward during the eyes open trial.

Secondary outcomes were the Timed Up & Go (TUG) test, hand grip strength, Falls Efficacy Scale (FES), and Falls Efficacy Scale-International (FES-I). The TUG test is a valid and reliable test of functional balance [31]. The participants started seated in a chair, and were then instructed to stand up and walk 3 m, turn around and walk back to the chair in their normal gait speed and sit down. The test was timed from when the participants were told to begin until they sat down again after performing the test. Validated Swedish translations of the FES-I [32, 33] and FES [34, 35] were used to measure rated fear of falling and balance confidence, respectively, before and after the intervention. The FES-questionnaire consists of 13 items with the score range 0–10, and a total score range of 0–130, where 130 indicates perfect confidence in performing daily activities without falling. The FES-I questionnaire consists of 16 items with the item score range 1–4 and the total score range 16–64, where 16 indicates no concern at all about the possibility of falling. Maximum isometric hand grip strength of the non-dominant hand was examined at baseline using a hydraulic hand dynamometer (Jamar; Patterson Medical, Warrenville, IL, USA). Participants were instructed to keep the arm at a 90° angle and to maintain the elbow in proximity to, but not pressed against, the waist while standing. The maximum value of two attempts was recorded.

### Randomization

Randomization was performed upon enrollment, using a six-sided dice for allocation at a 1:1-ratio between exercise and control. Balance exercise was allocated for faces 1–3, and no exercise was allocated for faces 4–6. The random allocation sequence, enrollment of participants, and intervention assignments were all handled by different personnel.

### Statistical analysis

Descriptive data are presented as means ± standard deviations, sums and percentages, or median and range for non-normally distributed data. Potential treatment effects were analyzed using ANCOVA with covariate-adjustments for baseline data [36]. 95% confidence intervals (CIs) of the mean change were calculated using the one-sample t-test with test value 0. Effect sizes (Cohen’s *d*) with 95% CIs were calculated for all outcome variables. All statistical analyses were performed in SPSS Statistics v24.0 (IBM Corporation, Armonk, NY, USA) on macOS v10.12.6 (Apple Incorporated, Cupertino, CA, USA) by an allocation-blinded researcher (co-author, unblinded after the 1st manuscript version was drafted). The significance level was set at 0.05.

## Results

### Participant flow

**Fig. 1:**
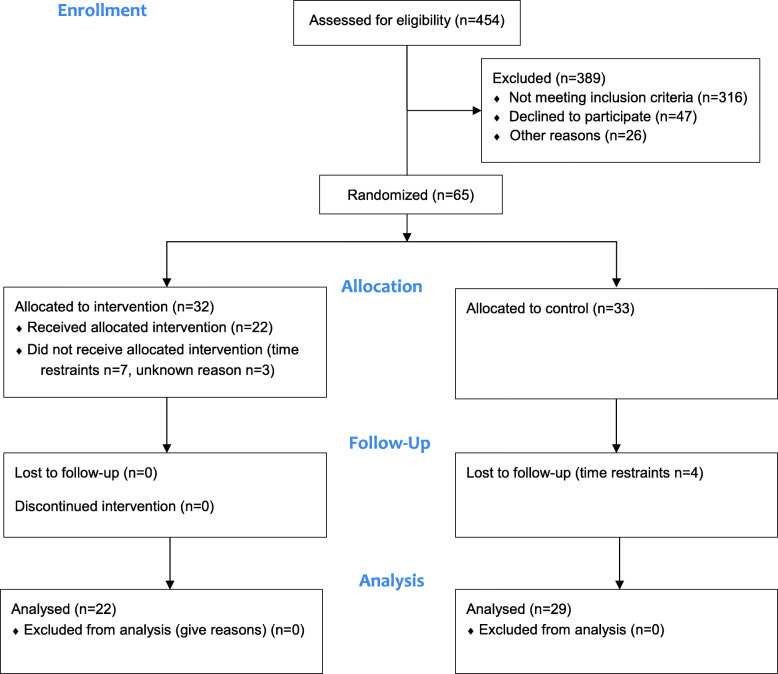
Participant flow chart

An overview of the participant flow is presented in Fig. 1. The original sample consisted of 454 community-dwelling individuals aged 70 years, drawn from the cohort study where they completed measurements of postural sway between November 14th, 2016 and May 12th, 2017. Of this original sample (*N* = 454), 143 individuals were identified meeting the inclusion criteria of PS_EO_ ≥ 400 mm or PS_EC_ ≥ 920 mm. Eligible individuals were contacted via telephone. Of these, 78 individuals were excluded from the trial because they did not meet the inclusion criteria of walking without a walking aid (*n* = 5), declined participation (*n* = 47), or did not respond on telephone (*n* = 26). After enrollment, 65 individuals had accepted participation and were randomly allocated to an exercise group (*n* = 32) or to a control group (*n* = 33). Between the allocation and trial onset, 10 participants in the exercise group dropped out because of time restraints (*n* = 7), or unknown reasons (*n* = 3). Between the allocation and follow-up, 4 control group participants dropped out due to time restraints during the scheduled follow-up assessments, leaving a total of 51 participants for intervention (*n* = 22) and control (*n* = 29) that completed the trial and were included in the outcome analysis. In the intervention group, the total mean attendance rate for the twelve group sessions was 87% (range, 67–100%), and 32% were fully adherent.

### Participant characteristics

Table 1 presents descriptive baseline data collected at the screening of participants before randomization. We observed that more than half of the participants in both groups had osteoarthritis, perceived vertigo or balance disorder, and had fallen during the previous year. The use of anti-hypertensives was of high frequency, and the total number of prescription drugs taken was high in both groups. The median number of prescription drugs being taken at baseline was *n* = 5 (exercise) and *n* = 4 (control), and the maximum was *n* = 16 (exercise) and *n* = 10 (control).

**Table 1:** Baseline characteristics and outcome variables

Characteristics	Intervention (***N*** = 22)	Control (***N*** = 29)
Men	14 (63.6%)	20 (69.0%)
Women	8 (36.4%)	9 (31.0%)
BMI	28.4 ± 6.6	28.2 ± 4.8
Smoker	2 (9.1%)	2 (6.9%)
Osteoarthritis	14 (63.6%)	16 (55.2%)
Vertigo / balance disorder	13 (59.1%)	15 (51.7%)
Sensory loss in feet or neck	6 (27.3%)	10 (34.5%)
Number of falls recent year	1 (0 to 10)	1 (0 to 4)
History of fall recent year	15 (68.2%)	17 (58.6%)
Anti-hypertensive use	15 (68.2%)	24 (82.8%)
Statin use	14 (63.6%)	11 (37.9%)
Total number of prescription drugs	5 (0 to 16)	4 (0 to 10)
*Outcome variables*		
PS_EO_ length (mm)	501.9 ± 112.9	486.9 ± 107.0
PS_EC_ length (mm)	1301.7 ± 749.1	1121.9 ± 579.7
Timed Up & Go (seconds)	11.2 ± 2.74	10.7 ± 2.28
Hand grip strength (kg)	28.5 ± 10.9	37.8 ± 10.3
FES (score)	127 (75 to 130)	129 (51 to 130)
FES-I (score)	21 (16 to 42)	18 (16 to 34)

Notes: Data are presented as N (%), means ± SD, or median (range)

*Abbreviations*: *PS* Postural sway, *BMI* Body mass index, *EO* Eyes open, *EC* Eyes closed, *TUG* Timed Up & Go, *FES* Falls Efficacy Scale, *FES-I* Falls Efficacy Scale International

### Outcomes

**Fig. 2:**
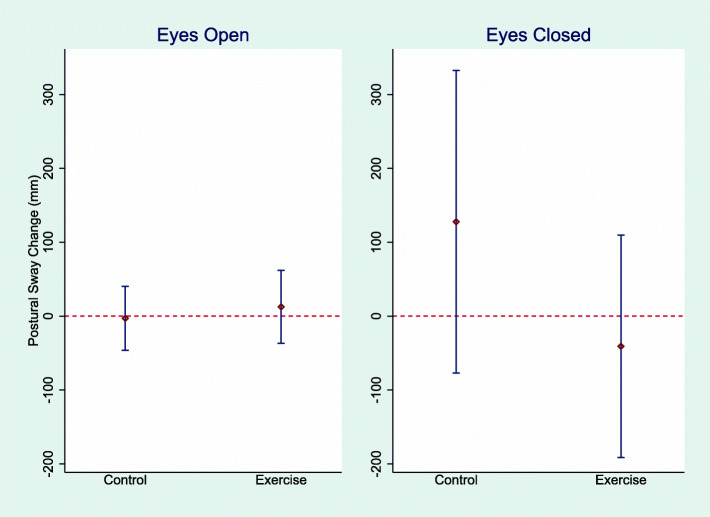
Mean change in postural sway outcomes. Notes: Data displayed are mean change and 95% confidence intervals for postural sway in the eyes open and eyes closed trials

Table 2 and Fig. 2 presents the analysis of the treatment effect and mean absolute change within the groups. No significant treatment effects were detected for primary or secondary outcomes. Within-group analyses showed that the intervention group increased their mean grip strength by 2.1 kg (95% CI 0.1 to 4.0) while the control group significantly reduced the mean TUG time by 1.2 s (95% CI − 2.0 to − 0.3). The participants reported no incidences of harm or adverse events during the trial, apart from delayed onset muscle soreness in the lower extremities.

**Table 2:** Mean change in outcomes with between-group comparisons

Outcomes	Intervention (***N*** = 22)	Control (***N*** = 29)	Cohen’s ***d***	***p***-value
PS_EO_ length (mm)	12.5 (−37.0 to 62.0)	−3.00 (−46.2 to 40.2)	0.21 (−0.34 to 0.77	0.57
PS_EC_ length (mm)	−41.0 (−191 to 110)	128 (−77.0 to 332)	0.02 (−0.53 to 0.57)	0.31
TUG (seconds)	−1.2 (−2.2 to 0.2)	− 1.2 (− 2.0 to − 0.3)*	0.22 (− 0.34 to 0.77)	0.61
Grip strength (kg)	2.1 (0.1 to 4.0)*	0.4 (− 1.1 to 1.9)	0.71 (0.14 to 1.28)	0.27
FES (score)	−0.2 (−4.9 to 4.5)	− 0.6 (− 2.9 to 1.64)	0.12 (− 0.43 to 0.67)	0.81
FES-I (score)	−0.7 (− 2.9 to 1.4)	0.8 (− 0.2 to 1.7)	0.15 (− 0.41 to 0.71	0.28

Notes: Data are presented as mean change from baseline (95% CI) and as effect sizes (Cohen’s *d*, 95% CI). Analyses of treatment effects were made using ANCOVA adjusted for baseline data. *statistical significance *p* < 0.05 for within-group changes

*Abbreviations*: *PS* Postural sway, *EO* Eyes open, *EC* Eyes closed, *FES* Falls Efficacy Scale, *FES-I* Falls Efficacy Scale International

## Discussion

The purpose of this study was to examine the effects of 4 weeks’ balance exercise on static and dynamic balance performance, fear of falling and self-efficacy, where the training was considered to be of initial low-threshold and progressively challenging suitable for untrained, older individuals. Previous studies have demonstrated that balance exercise programs lasting 6 weeks or more can be used effectively to reduce postural sway, increase TUG-performance, balance self-efficacy, and reduce fear of falling [16, 37]. The results of this four-week trial, however, showed no statistically significant and acute treatment effects on postural sway length, TUG time, FES-I score, or FES score, even though we included participants with documented high postural sway. Even so, we observed in the eyes closed trial that the intervention group reduced their mean postural sway length while the control group increased it, albeit no statistical significance was detected as previously mentioned. It has been argued that balance exercise influences PS_EC_ conditions more compared to PS_EO_ conditions, and that eyes closed trials exhibits a higher test-retest reliability (PS_EC_ ICC 0.94 vs. PS_EO_ ICC 0.60) [9, 16]. This can be explained by a more focused dependency on neuromuscular input during eyes closed conditions, a function that responds more to exercise compared to eyes open conditions that also involves visual input to regulate postural sway.

Both groups improved their TUG time by a mean of 1.2 s, although this change was only statistically significant in the control group. When compared with the results of previous analyses of the cohort, this decrease in TUG time translates to a 10.8% reduction in fall risk [9]. However, this improvement can potentially be explained by a practice effect from repeatedly performing the tests. Alternatively, the test’s normal pace condition could still have influenced participants to choose a quicker pace despite information from the test instructor. The intervention group significantly improved their handgrip strength by a mean of 2.1 kg, even though the balance exercise program was not designed to stimulate upper-body muscle strength. Others have reported associations between handgrip strength and balance ability [38], lending some support to the current study findings.

The intervention (Additional File 1) was purposely designed for a group setting to be appropriate for all participants with documented poor balance. The group-adjustment of exercise in combination with the heterogeneities in the participants’ functional status could have resulted in some individuals being sufficiently progressively challenged while others received a sub-optimal exercise volume. Thus, a more individualized approach to exercise could potentially have been favorable. The exercise volume of the intervention studied in this trial was ~ 45 min weekly over a four-week period, as the trial was designed to determine if positive effects on postural sway are achievable in a relatively short time in older individuals with poor balance. This design, if successful, could have had potential positive cost-effective and time-consuming implications. It is shown, however, that an exercise program lasting 11–12 weeks proved the most effective design to improve steady state balance in healthy young adults, while 6 weeks of combined strength and balance resulted in positive effects in healthy older adults [20, 21]. It therefore seemed reasonable that a 4-week exercise program could have positively influenced postural sway in balance-deficient older adults, as untrained individuals might gain exercise effects relatively quick [23]. The lack of short-term effects in the present trial may also indicate that higher weekly volumes of balance exercise is required. As such, it has been shown that weekly exercise volumes of ≥2 h over a 6-month period can improve fall rates [28], an outcome that has been linked to postural sway [9].

Another factor determining the outcome results of a balance exercise intervention pertains to how well the exercises are able to challenge the participants’ balance. It has previously been shown that exercise programs that offers the biggest challenge show the greatest reductions in falls [28]. The aim of the current intervention was to enable all participants to begin at a moderate level of difficulty and gradually increase the challenge to a higher level as they completed the different difficulty levels (Additional File 1). The levels of challenge are however subjectively perceived, as there are no standardized measures of exercise intensity in balance exercise trials, which makes it difficult to compare results, and the dose-response relationship of this type of exercise remains unclear. It is important to note that the participants anecdotally reported that the program was difficult to complete towards the end, and that some of the participants performed some of the exercises to failure. In addition, a previous study reported that balance exercise is highly task-specific and involves little transfer to other tasks that are not trained. This could potentially have affected the study outcomes even though we involved commonly used static exercises with different visual conditions to target bipedal postural stability, as well as dynamic exercises to target daily physical function as assessed by the TUG test [39]. It is also possible that the present study’s lack of significant effects were partly due to the exercise program not being able to produce an exercise stimulus that surpassed the reported minimal detectable change for the Nintendo Wii Balance Board [40].

Strengths of the present study lie in its RCT-design, blinded inclusion assessment and statistical analysis, and by the default age-adjusted data, since the study sample was drawn from a birth-cohort study. Furthermore, we included objectively measured postural sway as primary outcome, which holds advantages over clinical tests and has been shown to determine prospective fall risk in the same cohort that the current study sample was drawn from [9, 41]. The mean total compliance rate for the training sessions was 87%, which appears high when compared to the 53–79% reported by similar trials in an adjacent field [42].

### Study limitations

The lack of significant effects and the heterogeneity in the groups at baseline may be an indication of insufficient statistical power. For instance, the exercise group had lower handgrip strength and higher prevalence of comorbidities and medications. While we attempted to accommodate this by using ANCOVA to adjust for baseline data, we cannot entirely rule out that this potentially caused skewness in the results. In addition, we included individuals with poor balance regardless of underlying cause to increase generalizability of the results. While this can be perceived as a study strength, there is also a possibility that the higher prevalence of comorbidities and medication use influenced the 20% higher dropout rate in the intervention group, as they had to go through a challenging exercise regimen. Moreover, individuals were eligible for participation when meeting one of two independent inclusion criteria (PS_EO_, ≥ 400 mm and PS_EC_, ≥ 920 mm). Meeting both criteria were not mandatory since it would have entailed a smaller recruitment base, however, this could have contributed to a larger data heterogeneity. This study did not control for the time-of-day variances that has been shown to impact postural sway measurements [43], neither was the participant’s sleep status controlled for, another factor shown to affect postural sway especially in older individuals [44, 45]. In addition, baseline assessments for the primary outcome were drawn from the results of the cohort study up to 3 months ahead of eligibility assessment for this trial. Participants could potentially have altered their lifestyle during this period after participating in a larger health examination. It is however likely that potential lifestyle changes would be present in both participants of the intervention group and the control group.

## Conclusions

Results from the present trial suggest that a low-threshold and progressively challenging balance exercise program over a four-week period constitutes an insufficient dose to improve postural sway in balance-deficient participants. Individuals with high postural sway carries higher rates of comorbidities, such as neurological disorders and other factors that may interfere with the exercise response. Future balance exercise interventions should consider longer intervention periods than 4 weeks, a higher total exercise volume and adopt more individualized training protocols that take certain comorbidities and medications known to influence postural stability into account.

## Supplementary Information

**Additional file 1.**

## Data Availability

The datasets generated during and/or analysed during the current study are not publicly available due to regulations in the Swedish Personal Data Act, but may be made available from the corresponding author on reasonable request.

## Abbreviations

TUG: Timed Up & Go
FES: Falls Efficacy Scale
FES-I: Falls Efficacy Scale-International
PS_EO_: Postural sway length with eyes open
PS_EC_: Postural sway length with eyes closed
