# Supplementary material for: Short-term balance training and acute effects on postural sway in balance-deficient older adults: a randomized controlled trial
Source: BMC Sports Sci Med Rehabil. 2021 Mar 9;13:23. doi: 10.1186/s13102-021-00251-x (PMC7941938; doi:10.1186/s13102-021-00251-x)
Supplement: Supplementary file 1 — Additional file 1. [file 13102_2021_251_MOESM1_ESM.pdf]

Additional File 1 – Description of the intervention program

Title: Short-term balance training and acute effects on postural sway in balance-deficient older adults: A randomized controlled trial

Authors: Niklas Sörlén[a,c], Andreas Hult[b,c,g], Peter Nordström[d], Anna Nordström[c,e,g], Jonas Johansson[c,f,g]

Affiliations:

[a]Department of Clinical Science, Neurosciences, Umeå University, Umeå, Sweden
[b]Department of Community Medicine and Rehabilitation, Section for Sports Medicine, Umeå University, Umeå, Sweden
[c]Department of Public Health and Clinical Medicine, Section for Sustainable Health, Umeå University, Umeå, Sweden
[d]Department of Community Medicine and Rehabilitation, Geriatric Medicine, Umeå University, Umeå, Sweden
[e]School of Sport Sciences, UiT The Arctic University of Norway, Tromsø, Norway
[f]Department of Community Medicine, UiT The Arctic University of Norway, Tromsø, Norway
[g]Umeå School of Sport Sciences, Umeå University, Umeå, Sweden

Progression of static stances

	Level 1	Level 2	Level 3	Level 4	Level 5	Level 6
**Feet:**	Semi-tandem	Tandem	One foot	Semi-tandem	Tandem	One foot
**Vision:**	Normal	Normal	Normal	Eyes closed	Eyes closed	Eyes closed

- Colors symbolizes difficulty; green – easy, yellow – moderate, red - hard
- Participants were encouraged to stand for at least 10 and maximum 30 seconds in each stance. If 30 seconds was too easy, they were instructed to go up a level. If 10 seconds was too difficult, they were instructed to go down a level. Additionally, they were progressively encouraged to attempt releasing hold of the chair if this was used for support.
- Participants were instructed that the goal was not necessarily to reach level 6, but to continously challenge one self.

Instruction for feet positions:

Semi-tandem: Place one foot diagonally in front of the other and let the center of pressure rest on the back foot and use the front foot as support.

Tandem: Place one foot straight ahead of the other, let the center of pressure rest on the back foot and use the front foot as support.

One-leg: Stand on one leg, let the other foot rest just above the floor in the air, so that you can easily set it down if you feel that you are loosing your balance.

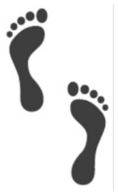

Semi-tandem

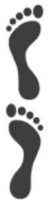

Tandem

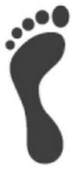

One-leg

Instructions for balance- and strength combined exercises

**Chair stands**<br>1. Sit down on a chair.<br>2. Stand up without using your hands.<br>3. Sit down in a controlled fashion.<br>4. Repeat at least 8-12 repetitions for 3 sets.<br><br>*If it was too difficult to perform this exercise without using hands, participants were instructed they could hold their hands against their knees or on the arm-support.*
**Side-kicks**<br>1. Stand behind a chair, with your hand on the back rest of the chair.<br>2. Lift your outer leg from the floor and move it with control out from the side of your body.<br>3. Move the leg back to the start position.<br>4. Repeat 8-12 times than switch leg.<br>5. Repeat for 3 sets for each leg<br><br>*Participants were encouraged to attemt lifting their hand slightly from the back rest of the chair, alternatively just use one or to fingers as support.*
**Calf raises**<br>1. Stand behind a chair with both your hands resting on the back of the chair as support.<br>2. Lift your heels upwards ands tand on your toes.<br>3. Move your heels down to the floor.<br>4. Repeat for 8-12 times and 3 sets.<br><br>*Participants were encouraged to try and reduce the hand support, either by just placing a finger or two on the chair, or remove the hands completely while performing the exercise.*
